# Evaluation of intraoperative MRI-assisted stereotactic brain tissue biopsy: a single-center experience in China

**DOI:** 10.1186/s41016-019-0152-0

**Published:** 2019-02-15

**Authors:** Chang-yu Lu, Zong-sheng Xu, Xun Ye

**Affiliations:** 1 Department of Neurosurgery, Peking University International Hospital, Beijing, China; 2 Department of Neurosurgery, Beijing Tiantan Hospital, Capital Medical University, Beijing, China

**Keywords:** High field-strength, Stereotactic technique, Brain biopsy, Intraoperative magnetic resonance

## Abstract

**Background:**

This study aimed to investigate the value of high field-strength intraoperative magnetic resonance imaging (iMRI)-guided stereotactic biopsy in the surgery of intracranial space-occupying lesions.

**Methods:**

A total of 87 patients who underwent stereotactic biopsy of intracranial lesions in the Peking University International Hospital from March 2016 to August 2018 were retrospectively surveyed; among these, 50 patients underwent MRI-guided stereotactic biopsy using the Leksell frame (iMRI group) and 37 cases received traditional stereotactic biopsy using the Leksell frame (control group). The accuracy rates and complications of the two groups were compared.

**Results:**

A 100% positive diagnosis was observed in all cases (*n* = 50) in the iMRI group. In 4 cases, the biopsy site was clearly found to have deviated from the target point, and the biopsy was performed again. The control group had 33 cases (86.5%) with positive diagnosis. No severe complications like neural functional deficit were observed in the iMRI group, while two patients developed bleeding at the puncture site (1 case receiving surgery to remove the hematoma) in the control group. There were no deaths in either group.

**Conclusion:**

iMRI-assisted stereotactic biopsy can confirm the target position and adjust the puncture path in real time. Compared to the traditional stereotactic biopsy technique, the iMRI method has a higher positive diagnostic rate, though surgical trauma and complications have no significant difference.

## Background

Traditional stereotactic brain biopsy (SBB) is a qualitative diagnostic technique, which allows pathological diagnoses and is widely used in clinical practice [1]. With the help of navigation devices, patients are now increasingly subject to intracranial lesion biopsies. The stereotactic guided biopsy has several advantages including patients unnecessary to receive general anesthesia. In addition, it leads to a short surgery time and an easy operation process, thus having an irreplaceable role.

The traditional SBB technique takes advantage of stereotactic orientation and uses the stereotactic instrument to intracranially guide the biopsy needle. It developed rapidly and is widely used due to the ease of operation [2, 3]. The key step of the surgery is to reach the diseased tissue while avoiding important functional structures. SBB is an invasive surgery, which sometimes induces complications, such as intracranial hemorrhage, epilepsy, infection, and tumor implantation. Malone et al. analyzed 7514 SBB cases and found a complication rate of 6.1%, with a bleeding rate of 5.8% [4]. In order to avoid bleeding after puncture, the operation should avoid vascular-rich parts, such as the sulcus and sinus. Using the stereotactic surgery system software and image fusion technology, the target can be quickly and automatically calculated. The multi-source image fusion and 3D reconstruction techniques provide an intuitive and accurate indication of the lesion and the surrounding tissue structures. Although traditional stereotactic biopsy using preoperative image data has been considered safe and effective, the indirect view of the framed stereotactic operation is limited in tracking the intracranial condition in real time. In addition, when the dura is opened, the cerebrospinal fluid loss and biopsy needle implantation may lead to intraoperative brain shift and sampling deviation from the target site, which results in a reduced positive rate.

The continued evolution of image-guided surgical techniques over the past 20 years led to tremendous advances in the field of neurosurgery [5], by allowing high field-strength intraoperative magnetic resonance imaging (iMRI) scans and updated image data to be obtained at any time. The techniques enable surgeons to image the patient via an MRI scanner while the patient is undergoing surgery, particularly brain surgery. Neurosurgeons rely on iMRI technology to create accurate pictures of the brain that guide them in removing brain tumors and other abnormalities during operations. To utilize iMRI technology during surgery, surgeons use portable iMRI devices or nearby iMRI devices as they need to check the accurate position of abnormalities. When and how often iMRI images are created during surgery depends on surgical procedure and patient’s condition. This integrating technology allows the surgeon to update the surgical plan in real time, compared to the preoperative plan. In this study, iMRI facilitates an accurate guidance of the biopsy operation and reduces severe complications and secondary damage to the important functional structures near the target.

## Methods

### Patients

We retrospectively analyzed 87 patients with stereotactic brain biopsy performed at the Peking University International Hospital from March 2016 to August 2018. The iMRI group included 50 cases (30 males and 20 females), with an average age of 43.5 ± 5.7 years. The lesions were located at the frontal lobe for 20 cases, at the temporal lobe for 14 cases, at the parietal lobe for 6 cases, at the insular-basal ganglia for 5 cases, at the thalamus for 3 cases, and at the midline (carcass and saddle) for 2 cases. The control group included 37 patients (25 males and 12 females) with an average age of 47.3 ± 7.9 years. The lesions were located at the frontal lobe for 19 cases, at the temporal lobe for 4 cases, at the parietal lobe for 6 cases, at the insular-basal ganglia for 4 cases, at the thalamus for 3 cases, and at the midline (carcass and saddle area) for 1 case.

### Scan protocols

For the iMRI group, all cases were scanned by normal MRI 1 day before biopsy. The scan data were stored in the DICOM format, including the T1-weight sequence, T2-weight sequence, T2flair-weight sequence, enhanced 3DT1-weight sequence, and DTI-weight sequence. An MRA sequence besides the fMRI image was acquired if necessary.

The iMRI scan data were transmitted to the navigation workstation for image fusion. Next, the lesion range was delineated, and the lesion was displayed in a three-dimensional mode. For enhancement, the lesion profile was depicted using the 3DT1 sequence, or else the lesion range was delineated with the T2 or T2flair sequence. Corticospinal tract (CST) reconstruction was performed for the motor activation region when necessary. The system revealed the 3D anatomical relationships among the lesion, the CST, and the hand movement activation areas. (The hand movement activation area refers to the cortical and subcortical fiber conduction bundles closely related to the hand movement function, which is an “Ω”-shaped “knot area” which is bent and folded back toward the central groove, which makes the anterior central gyrus area enlarged several times, and the white matter fiber conduction beam is denser in this area. This is compatible with the most delicate and complex hand movements in the body movement. The anatomical and functional characteristics of the hand movement area are proposed for the higher requirement in this area’s operation.) Based on these, we designed the biopsy path using the BrainLab workstation and developed the optimal surgical plan. The principles of biopsy path selection were as follows: avoiding the ventricle, the important functional area of the cerebral cortex, the CST, and the main vascular areas, passing through the center of the lesion, particularly for multiple targets. The patient was placed in the iMRI operating room under local anesthesia, and the Leksell-G stereotactic pedestal base and frame were installed. The center of the frame was located around the lesion. We selected the appropriate position, routinely disinfected the drape, and installed the positioning bow and guide biopsy system according to the *X*, *Y*, *Z* coordinate values and the arc and ring angles. After scalp exposure, the sharp knife pierced the skin, and the skull was drilled with a 3-mm-diameter micro-drill. Blunt dilatation was performed after the dura was pierced. The lateral cutting biopsy needle was used for sampling. The sample was strip of size 2 mm × 10 mm. Each target was sampled in two to three pieces. The tissue was fixed by 10% formaldehyde. After scalp suture or pressure wrapping, the bow frame was removed, and the iMRI scan was performed again to compare with the preoperative images and ensure that the puncture orbit reached the target without complications such as bleeding. The specimen was sent for pathological examination. If the intraoperative scan indicated an obvious deviation from the target point, the scan was performed again (the positioning bow and the guided biopsy system were re-installed) and the surgical plan was updated in real time according to the iMRI image.

For the control group, the preoperative scan sequences and surgical plan were identical to the iMRI group. After biopsy, the needle and the frame were removed. The scalp was sutured or pressure-wrapped, and the specimen was sent for pathological examination.

### Statistical analyses

All data were analyzed using SPSS 22.0. Quantitative data are expressed as mean ± standard deviation. Student’s *t* test or Fisher’s exact test was used to compare the differences between the two groups. The multivariate statistical analysis was used in biopsy results with pathology or location adjusted. A *P* value less than 0.05 was considered statistically significant.

## Results

Patients’ clinical characteristics in the study are summarized in Table 1; there was no significant difference in age (i.e., *P* > 0.05) between the two groups except for different treatment. Complications such as neural functional deficit, intracranial hemorrhage, postoperative bleeding, epilepsy, diffuse brain edema, and tumor implantation were considered severe complications, and others like operative site infection and wound breakdown were considered minor complications.

**Table 1:** Patient clinical characteristics

Parameter	iMRI (*n* = 50)	Control (*n* = 37)	Fisher’s exact test	*P*
Sex				
Male	30 (60.0%)	25 (67.6%)	0.524	0.469
Female	20 (40.0%)	12 (32.4%)		
Age	45.3 ± 5.7	47.3 ± 7.9	− 1.372	0.174
Bleeding				
Yes	0 (0.0%)	2 (58.8%)		0.178
No	50 (100.0%)	35 (41.2%)		
Lesion region				
Frontal lobe	20 (40.0%)	19 (51.4%)	4.411	0.492
Temporal lobe	14 (28.0%)	4 (10.8%)		
Parietal lobe	6 (12.0%)	6 (16.2%)		
Insular-basal ganglia	5 (10.0%)	4 (10.8%)		
Thalamus	3 (6.0%)	3 (8.1%)		
Midline	2 (4.0%)	1 (2.7%)		

In 46 of the 50 patients in the iMRI group, the puncture orbit was confirmed to have reached the target site and the biopsy was successful. In the other 4 patients, it was found that the site had deviated from the target, and the procedure was performed again. The results of pathological diagnosis were as follows: 26 cases of glioma, 2 cases of germ cell tumor, 17 cases of lymphoma, 1 case of inflammation, 1 case of abscess, and 3 cases of metastases. No postoperative bleeding occurred in the iMRI group.

In the traditional framed (control) group, a positive diagnosis was obtained in 33 cases. The pathological findings were as follows: 17 cases of glioma, 12 cases of lymphoma, 1 case of demyelinating disease, 1 case of metastasis, 1 case of radioactive necrosis, and 1 case of schwannoma. Two patients developed hemorrhage at the biopsy site, including one with a hemorrhage volume > 30 mL who received emergency craniotomy and hematoma evacuation combined with tumor resection. Though there were no significant differences in Table 1, we found much more advantages in iMRI treatment as there were no severe complications occurred. There was no new neurological function damage before discharge. The other hemorrhage patient received conservative observation and hematoma absorption. There were no deaths or postoperative infections in either group. Based on the pathological results, patients underwent surgery, radiotherapy, chemotherapy, anti-inflammatory therapy, or other clinical procedures.

**Fig. 1:**
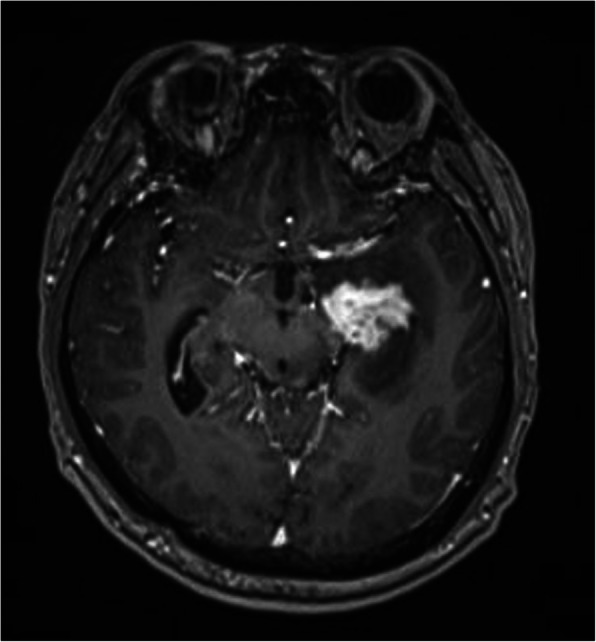
Multiple intracranial neoplastic lesions were indicated by enhanced MRI and magnetic resonance spectroscopy imaging, probably lymphoma

**Fig. 2:**
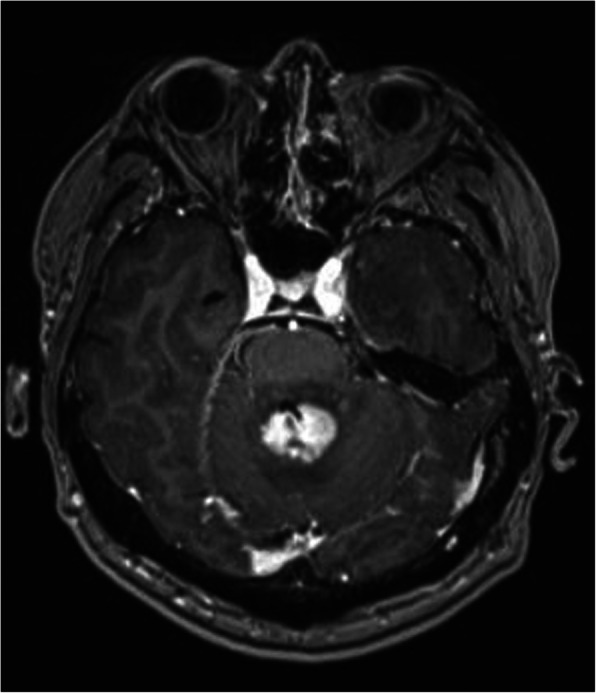
Multiple intracranial neoplastic lesions were indicated by enhanced MRI and magnetic resonance spectroscopy imaging, probably lymphoma

**Fig. 3:**
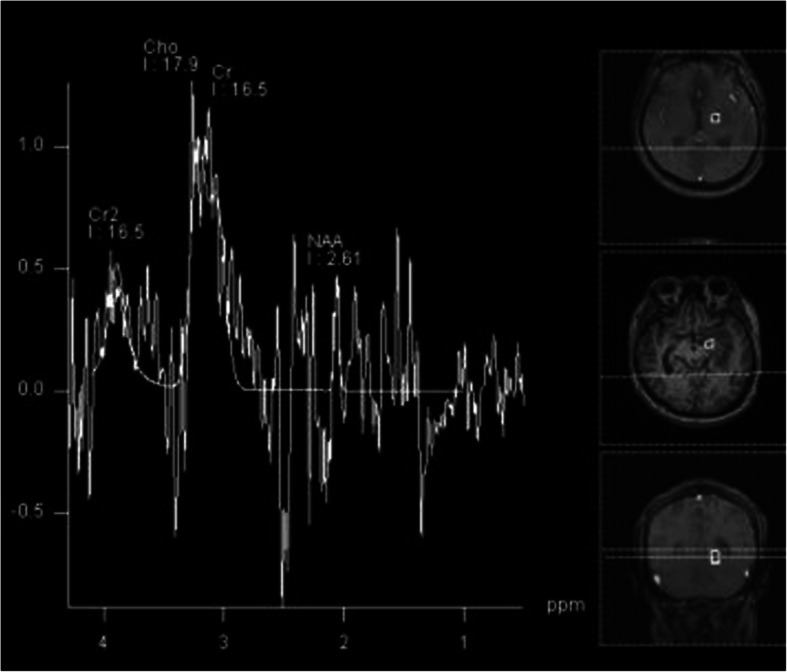
Multiple intracranial neoplastic lesions were indicated by enhanced MRI and magnetic resonance spectroscopy imaging, probably lymphoma

**Fig. 4:**
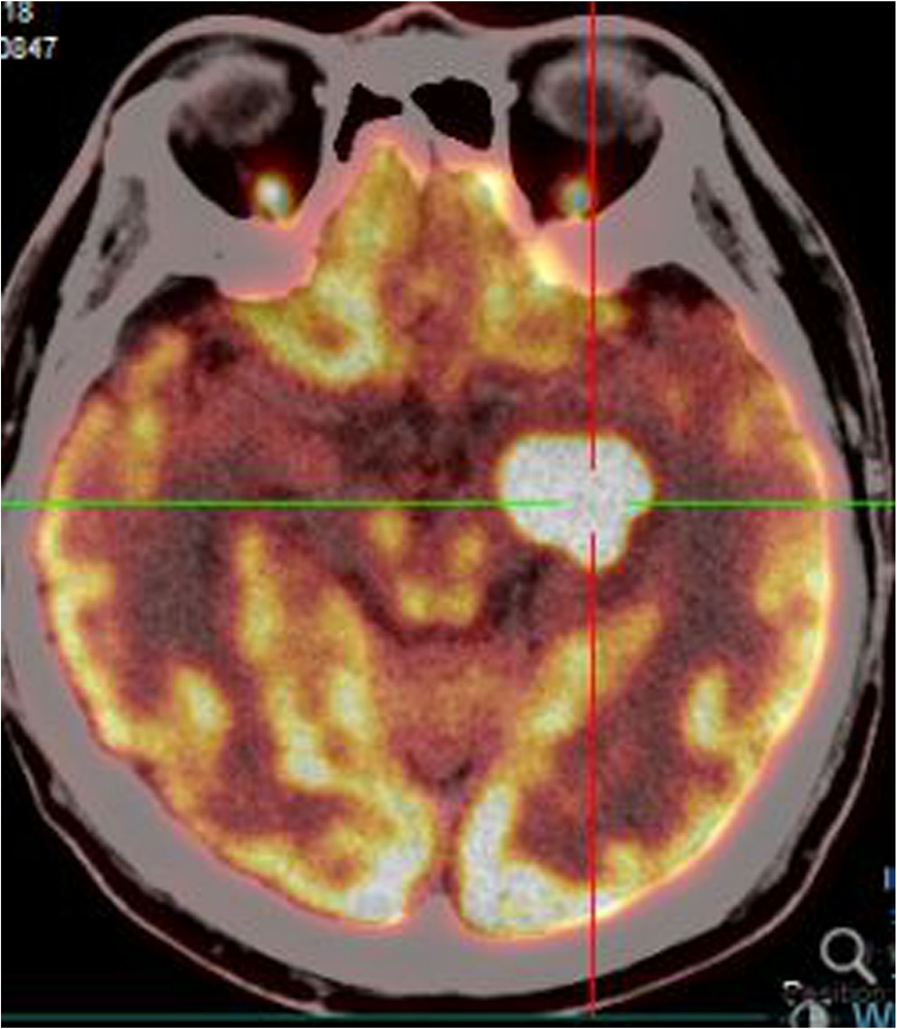
PETCT scan showed a high-level metabolism in the lesion areas

**Fig. 5:**
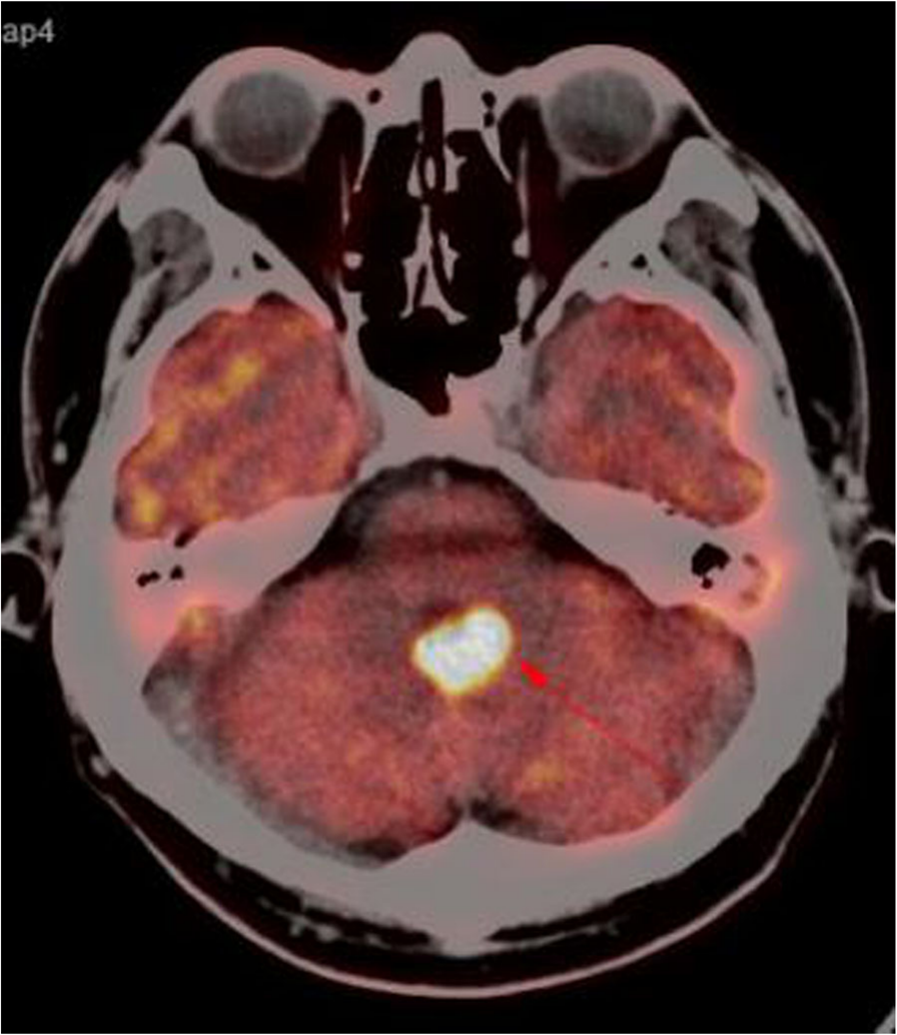
PETCT scan showed a high-level metabolism in the lesion areas

**Fig. 6:**
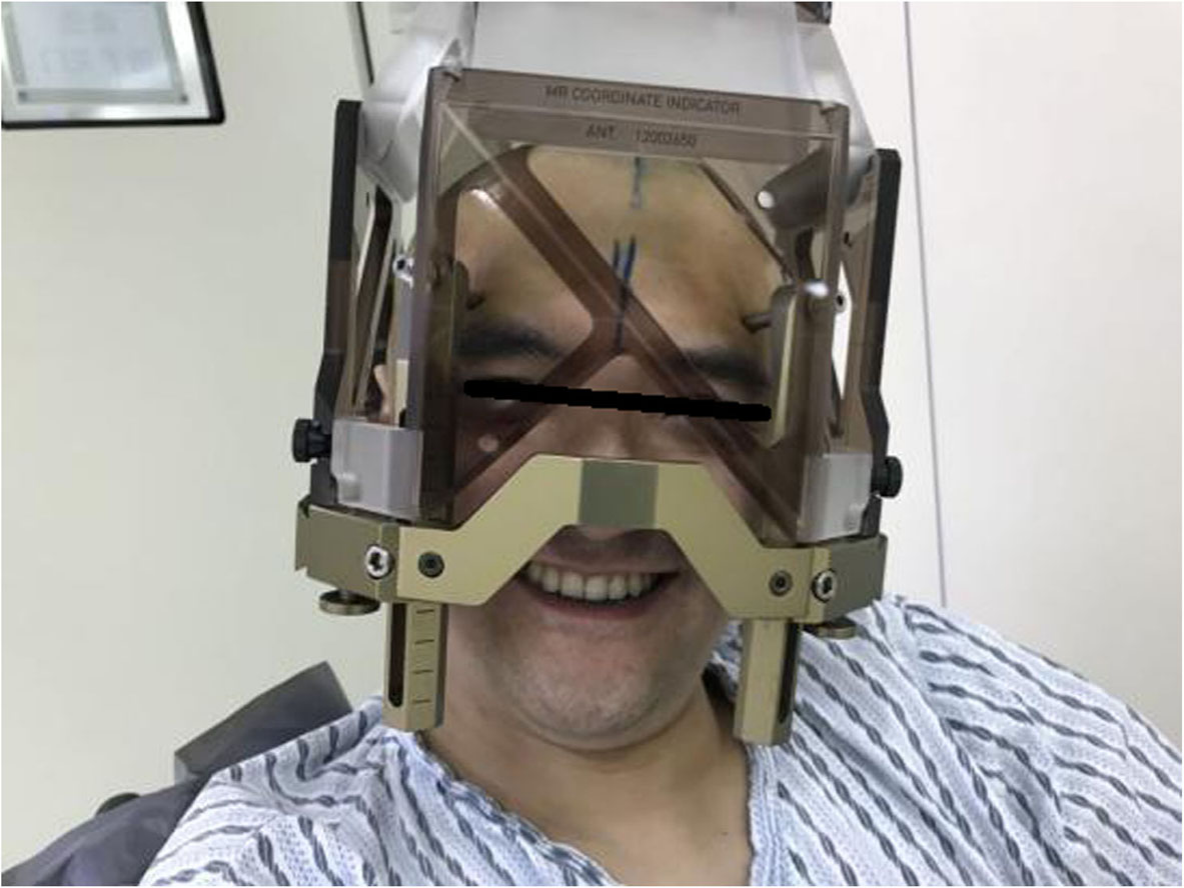
MRI was performed to locate the lesions after preoperative installation of the leksell headgear

**Fig. 7:**
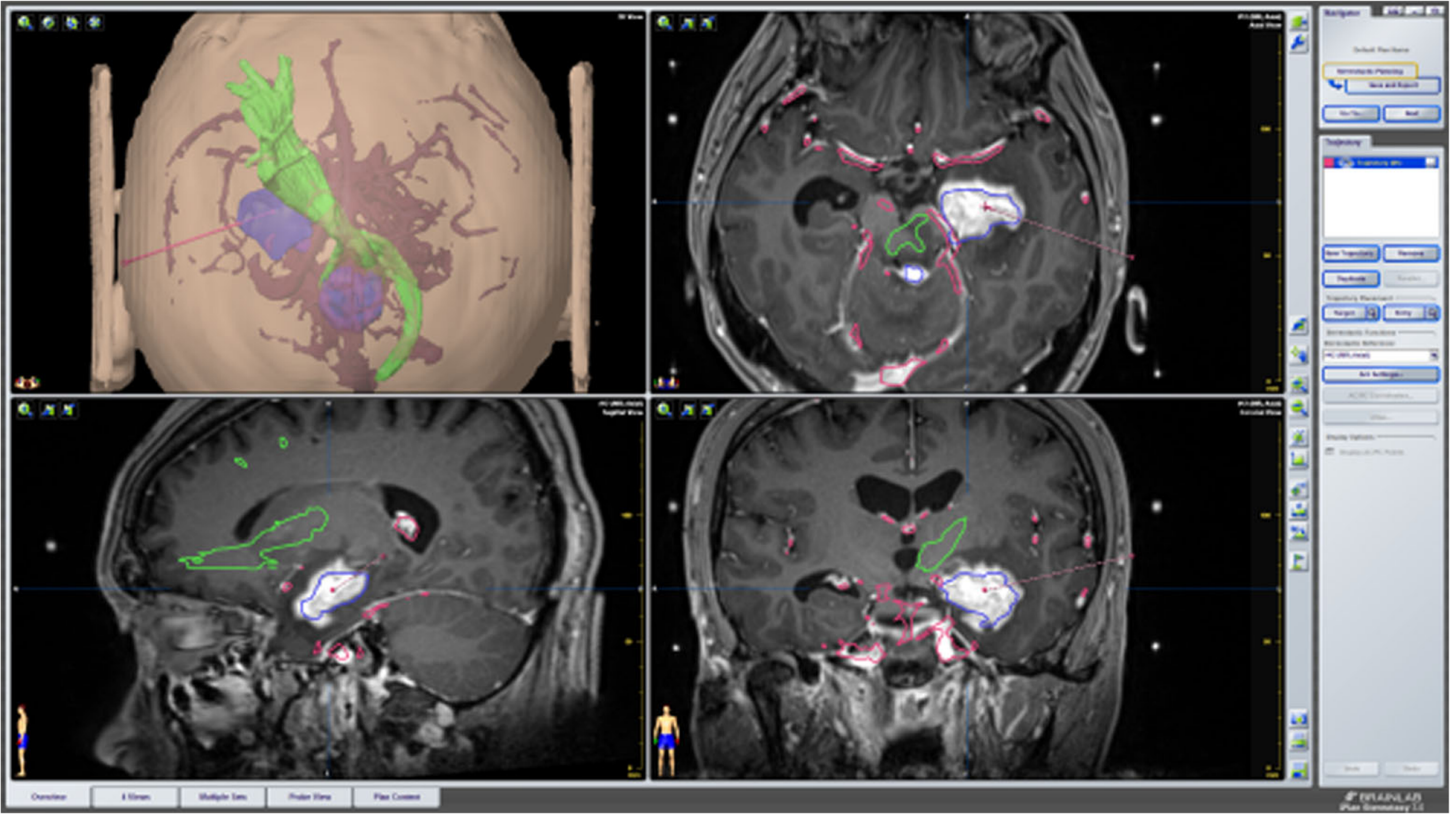
Multi-image fusion and three-dimensional reconstruction showed the relationship between lesions (upper left in blue) and fiber bundles (upper left in green) as well as intracranial vessels (upper left in maroon)

**Fig. 8:**
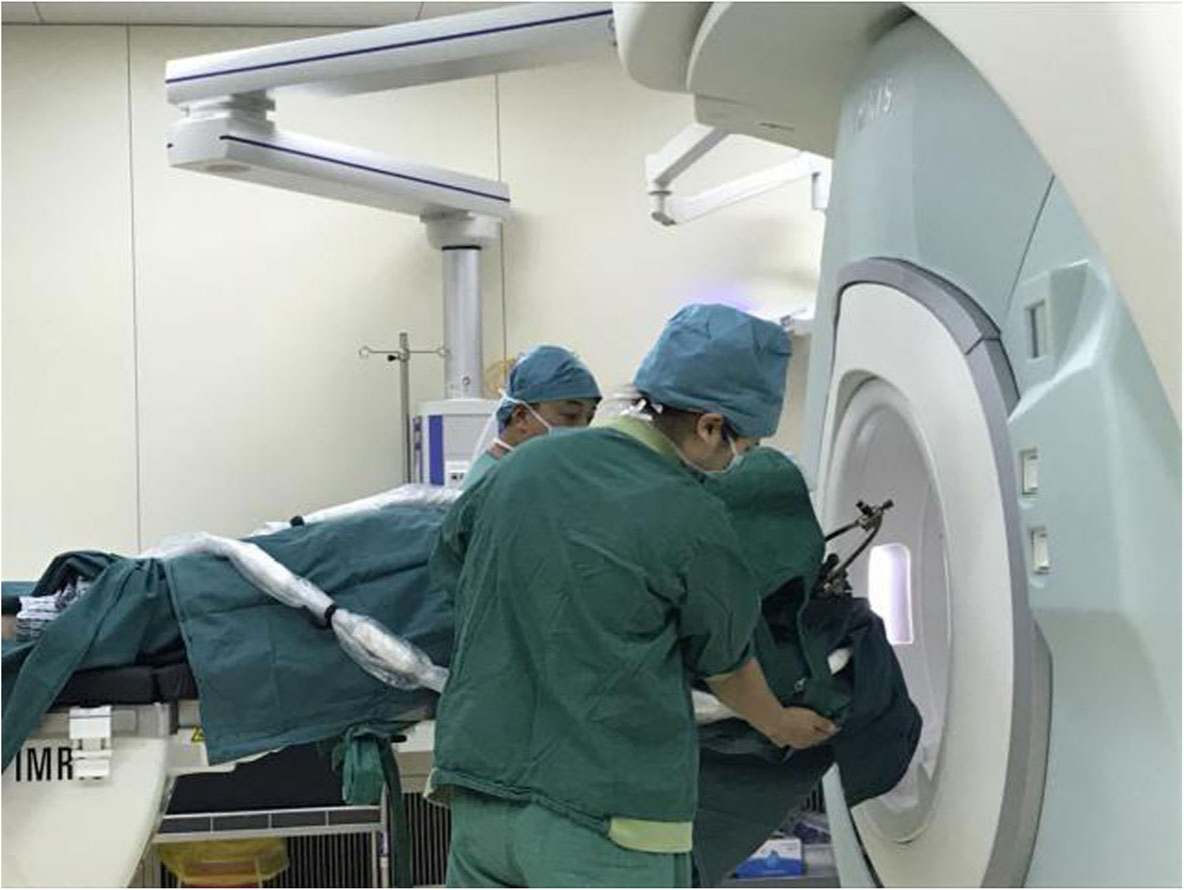
The patient underwent an iMRI scan

**Fig. 9:**
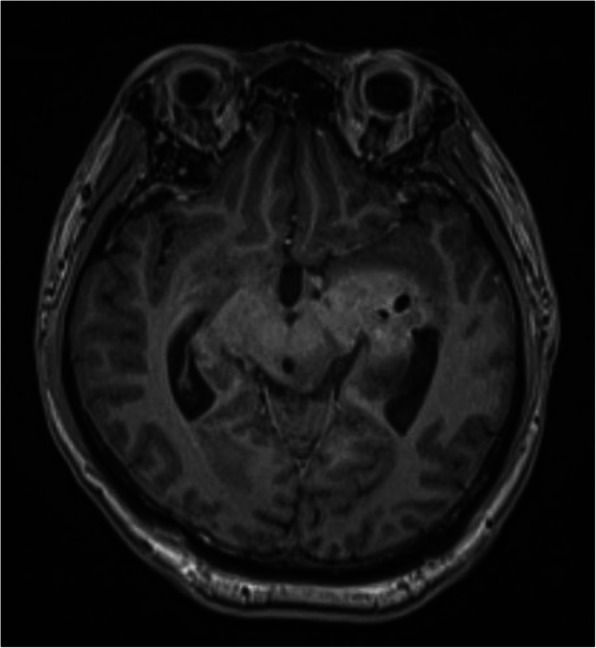
Intraoperative MRI confirmed no secondary bleeding. In addition, the puncture orbit was consistent with the preoperative planned orbit with no deviation, and the target lesion was weakened and the specimen was successfully obtained

**Fig. 10:**
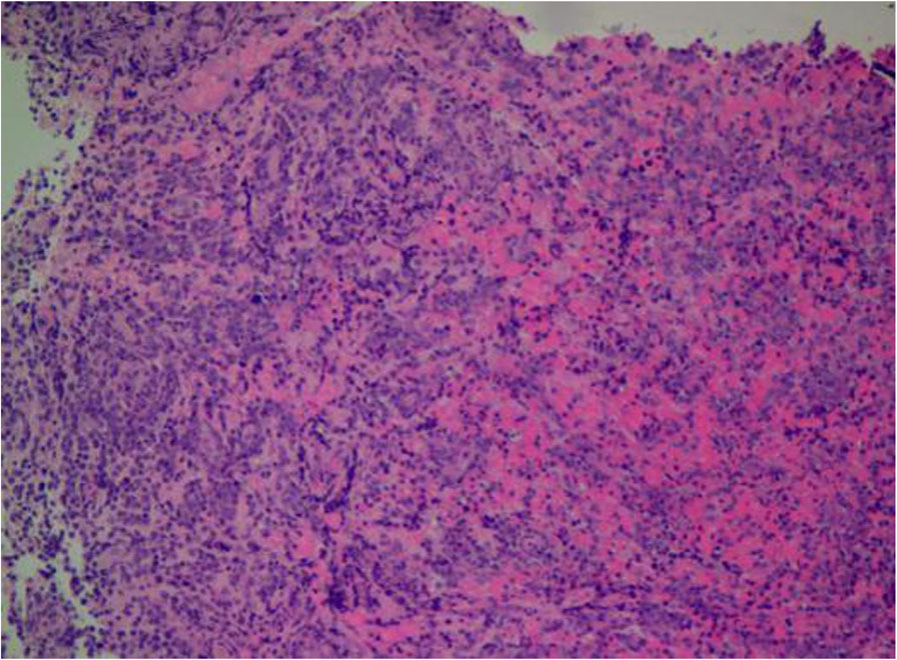
Postoperative pathological analysis revealed a non-Hodgkin lymphoma and diffuse large B-cell lymphoma. Immunohistochemical staining showed CD3(-), CD20(+), PAX5(+), Ki-67 (about 70%+), CD10(-),Bcl-6(+), Mum-1(+), BCL2 (about 80%+), C-MYC (about 40%+), CD5(+), CD21(-), CyclinD1(-), CK(-), GFAP(-)

A typical case: a 38-year-old right-handed male was hospitalized mainly due to progressive left limb fatigue for more than 3 months and aggravated nearly 1 week. Physical examination showed a poor coordinate movement of the left limbs. Enhanced MRI and magnetic resonance spectroscopy imaging indicated multiple intracranial neoplastic lesions, probably lymphoma (Figs. 1, 2, and 3). PETCT scan showed a high-level metabolism in the lesion areas (Figs. 4 and 5). MRI was performed to locate the lesions after preoperative installation of the Leksell headgear (Fig. 6). Preoperative surgical plan was conducted at the BrainLab workstation. Multi-image fusion-guided stereotactic surgery was performed for intracranial lesion biopsy. Multi-image fusion and three-dimensional reconstruction showed the relationship between lesions and fiber bundles as well as intracranial vessels (Fig. 7). Based on the biopsy target and the path, the biopsy needle was placed into the lesion center, and the specimen was obtained after multidirectional suction at a single target. After surgery, the patient underwent an iMRI scan (Fig. 8). Intraoperative MRI confirmed no secondary bleeding. In addition, the puncture orbit was consistent with the preoperative planned orbit with no deviation, and the target lesion was weakened and the specimen was successfully obtained (Fig. 9). Postoperative pathological analysis revealed a non-Hodgkin lymphoma and diffuse large B cell lymphoma. Immunohistochemical staining showed CD3(−), CD20(+), PAX5(+), Ki-67 (about 70%+), CD10(−), Bcl-6(+), Mum-1(+), BCL2 (about 80%+), C-MYC (about 40%+), CD5(+), CD21(−), CyclinD1(−), CK(−), and GFAP(−) (Fig. 10). After the operation, the patient's symptoms were not aggravated, and had no other neurological dysfunction.

## Discussion

Since Spiegel and Wycis applied stereotactic technology in human beings in 1947, this technique has rapidly developed and has become widely used with the improvement of computer and imaging technologies. Stereotactic biopsy has become a basic operation. It has the advantages of micro-invasiveness and high diagnostic positive rate and has almost replaced the previously used craniotomy and experimental treatment. To date, stereotactic biopsy is one of the most important methods for the diagnosis of difficult diseases in Neurosurgery departments. Intracerebral lesion biopsy can be divided into two types: framed and frameless. It was reported that there were no statistically significant differences in positive and complication rates between the two surgical methods. The biopsy technique using the Leksell stereotactic instrument is very simple, with low operating costs, and is therefore widely used [6–9]. This technique is especially useful for patients presenting lesions in important functional areas, multiple intracranial lesions, and extensive lesions and those with difficult surgical resections. Some types of tumors, such as gliomas and lymphomas, are sensitive to chemo-radiotherapy, and craniotomy is not recommended. With the support of modern computer and imaging technology, stereotactic orientation is very safe but accurate (the error is less than 2 mm) [10]. Dammers et al. [11] reported a total of 12,038 cases from 2001 to 2010 and showed that the positive rate of biopsy diagnosis was 94.7% (83.6–100%), the postoperative complication rate was 4.7% (0.7–16.1%), and the case fatality rate was 0.9% (0–3.9%). Traditional framed stereotactic biopsy is based on preoperative images. Once brain shift occurs during surgery, the accuracy is lost. Intraoperative imaging techniques such as iMRI are effective means to solve intraoperative brain shifts. There are few reports on iMRI-assisted frame-type brain stereotactic biopsy. With the iMRI-assisted stereotactic needle biopsy, the position and angle of the needle can be continuously adjusted through the intra-operatively updated image, until the needle is located inside the lesion. This real-time visualization of the biopsy greatly enhanced the positive rate of pathological diagnosis [12–14]. Sawin et al. [15] argued that the more biopsy samples are obtained, the greater the probability of postoperative complications. Using iMRI navigation, we can reduce the amount of sampling as shown in the results compared with previous studies [16] and thus reduce the probability of postoperative complications. In addition, the postoperative iMRI scan can detect complications (e.g., early bleeding) in time. It was even pointed out that the iMRI navigation needle biopsy can avoid frozen section analysis.

In our study, the positive rate of biopsy in the iMRI group was 100%, higher than that in the control group which is 86.5% (Table 2). There were 4 cases (8%) in the iMRI group found deviated from the preoperative target. After the navigation update and second puncture, the iMRI scan confirmed that these 4 cases of error sites were corrected. Another advantage of iMRI is the timely discovery of complications (e.g., intraoperative bleeding), largely improving the safety of surgery. In this study, two cases of hematoma which were considered as severe complications were found in the control group, and one of them underwent craniotomy. The iMRI examination can detect bleeding in the biopsy site in time, which is beneficial to the patient by allowing timely decisions.

**Table 2:** Diagnostic results

Results	iMRI (*n* = 50)	Control (*n* = 37)	Fisher’s exact test	*P*
Positive	50 (100.0%)	32 (86.5%)	4.891	0.027
Negative	0 (0.0%)	5 (13.5%)		

Nonetheless, the sample size of this study is small, and we have only described preliminary clinical application experience. In future studies, we plan to involve a large sample of randomized controlled trials to compare the differences between biopsy using iMRI and traditional framed biopsy in terms of positive rate and postoperative complication rate.

In summary, the main advantage of iMRI navigation in biopsy is the possibility to correct in real time for intraoperative brain shifts, thus improving the positive rate of biopsy and reducing the incidence of postoperative complications. In addition, the cost of a biopsy is lower than that of general anesthesia, and the operation time under iMRI navigation is shorter. It is reasonable to believe that its application is promising in future clinical applications.

## Conclusions

The iMRI-assisted stereotactic biopsy could help to position the target and adjust the puncture path in a real-time way. Compared to the traditional frame stereotactic biopsy, a higher positive rate in diagnosis can be acquired using this protocol.

## Abbreviations

CST: Corticospinal tract
iMRI: Intraoperative magnetic resonance imaging
SBB: Stereotactic brain biopsy
